# Transcriptome and Gene Ontology (GO) Enrichment Analysis Reveals Genes Involved in Biotin Metabolism That Affect l-Lysine Production in *Corynebacterium glutamicum*

**DOI:** 10.3390/ijms17030353

**Published:** 2016-03-09

**Authors:** Hong-Il Kim, Jong-Hyeon Kim, Young-Jin Park

**Affiliations:** Department of Biomedical Chemistry, Konkuk University, Chungju 27478, Korea; kwangdae7@kku.ac.kr (H.-I.K.); dhqksdl@kku.ac.kr (J.-H.K.)

**Keywords:** biotin, *Corynebacterium glutamicum*, l-lysine, transcriptome, gene ontology (GO) term enrichment

## Abstract

*Corynebacterium glutamicum* is widely used for amino acid production. In the present study, 543 genes showed a significant change in their mRNA expression levels in l-lysine-producing *C*. *glutamicum* ATCC21300 than that in the wild-type *C*. *glutamicum* ATCC13032. Among these 543 differentially expressed genes (DEGs), 28 genes were up- or downregulated. In addition, 454 DEGs were functionally enriched and categorized based on BLAST sequence homologies and gene ontology (GO) annotations using the Blast2GO software. Interestingly, NCgl0071 (*bioB*, encoding biotin synthase) was expressed at levels ~20-fold higher in the l-lysine-producing ATCC21300 strain than that in the wild-type ATCC13032 strain. Five other genes involved in biotin metabolism or transport—NCgl2515 (*bioA*, encoding adenosylmethionine-8-amino-7-oxononanoate aminotransferase), NCgl2516 (*bioD*, encoding dithiobiotin synthetase), NCgl1883, NCgl1884, and NCgl1885—were also expressed at significantly higher levels in the l-lysine-producing ATCC21300 strain than that in the wild-type ATCC13032 strain, which we determined using both next-generation RNA sequencing and quantitative real-time PCR analysis. When we disrupted the *bioB* gene in *C*. *glutamicum* ATCC21300, l-lysine production decreased by approximately 76%, and the three genes involved in biotin transport (NCgl1883, NCgl1884, and NCgl1885) were significantly downregulated. These results will be helpful to improve our understanding of *C*. *glutamicum* for industrial amino acid production.

## 1. Introduction

*Corynebacterium glutamicum* is widely used for the biotechnological production of industrially important amino acids, such as l-glutamate and l-lysine [1,2]. After discovering that *C*. *glutamicum* can secrete amino acids, many researchers have attempted to develop an industrial strain by a classical random mutagenesis approach [3]. However, mutants derived from random mutagenesis are generally inferior to their wild-type strains concerning industrially important properties, such as their growth, sugar consumption, and stress tolerance. These limitations have generally restricted the establishment of highly productive industrial strains [1]. Therefore, to systematically develop mutants that avoid these limitations, various approaches, including the quantitative assessment of metabolic fluxes, enzymatic and signaling pathway analyses combined with metabolite pool determination, and functional genomic technologies (e.g., DNA microarrays), were performed [4,5,6]. Indeed, genetic engineering methods have been utilized to alter (*i.e.*, mostly to increase) the expression of genes-of-interest to construct *C*. *glutamicum* strains that can produce and secrete commercially important amino acids [7,8,9,10]. In theory, new targets for modulating gene expression can now be rapidly identified with global gene transcriptional profiling (e.g., DNA microarrays) methods to further develop industrially relevant strains [5,6].

Recently, next-generation sequencing (NGS)-based technologies have reduced the cost and time of genome-wide analysis. Thus, we compared the global mRNA expression of l-lysine-producing *C*. *glutamicum* ATCC21300 and wild-type *C*. *glutamicum* ATCC13032 using next-generation RNA sequencing (RNA-seq). Our findings provide insight into the general physiology of the cells, specific amino acid production mechanisms in *C*. *glutamicum,* and potential advances in the rational engineering of industrially advantageous strains.

## 2. Results

### 2.1. Identification of Differentially Expressed Genes and Gene Ontology (GO) Enrichment Analysis

**Figure 1:**
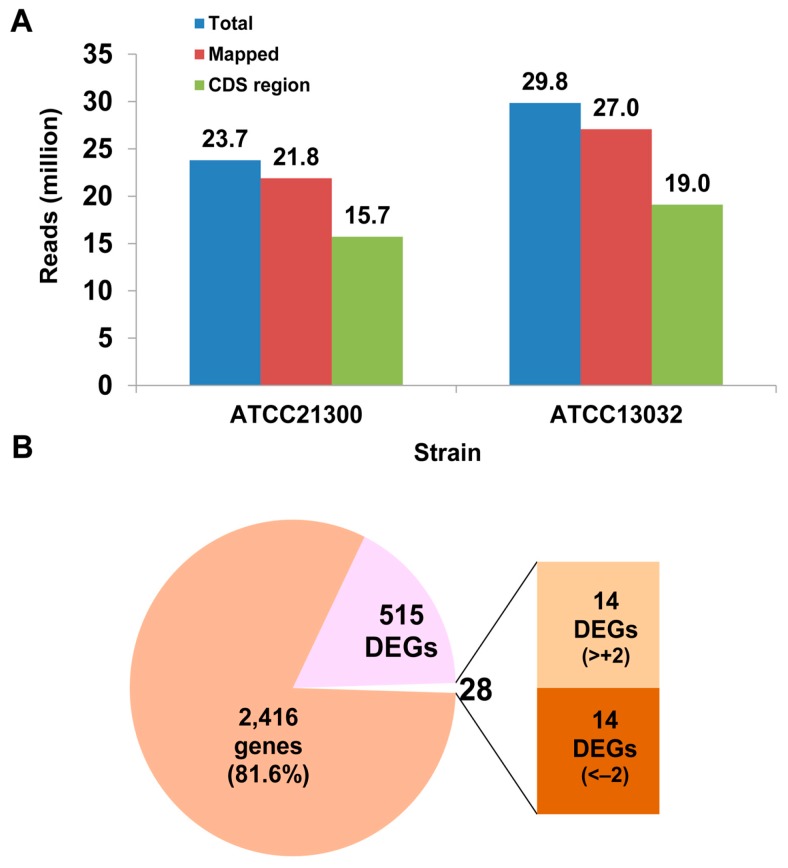
Characterization of global transcriptome in *Corynebacterium glutamicum* ATCC21300 and ATCC13032. (**A**) The total number of mRNA-Seq (sequencing) reads mapped in each *C. glutamicum* strain library; and (**B**) differentially expressed genes (DEGs) in *C. glutamicum* ATCC21300. Detailed information is shown in Table S2. CDS: coding sequencing.

To compare the transcriptome of l-lysine-producing *C. glutamicum* ATCC21300 and wild-type *C. glutamicum* ATCC13032, RNA sequencing libraries were constructed and short reads were verified. A total of 23,799,828 and 29,850,918 reads, generated from *C. glutamicum* ATCC21300 and ATCC13032 libraries, respectively, were mapped to the *C. glutamicum* ATCC13032 genome sequence (National Center for Biotechnology Information (NCBI) reference sequence: NC_003450); 66.2% and 63.7% of reads, respectively, were matched to annotated CDS (coding sequencing) regions (Figure 1A).

We performed statistical analysis using DEG-Seq to establish that mRNA expression levels of 543 genes differed significantly (*p* < 0.001 in *t*-tests) between *C. glutamicum* ATCC21300 and ATCC13032. Among these 543 genes, the expression of 28 genes were dramatically different (>+2 or <−2 log2 fold change) (Figure 1B and Table S2). In *C*. *glutamicum*, 11 genes are involved in l-lysine biosynthesis. Among them, 9 genes (NCgl0247; *lysC*, NCgl0248; *asd*, NCgl1058; *dapC*, NCgl1061; *dapD*, NCgl1064; *dapE*, NCgl1868; *dapF*, NCgl1896; *dapA*, NCgl1898; *dapB*, and NCgl2528; *ddh*) were slightly upregulated and 2 genes (NCgl1133; *lysA* and NCgl1214; *lysE*) were slightly downregulated in *C*. *glutamicum* ATCC21300 compared to in *C*. *glutamicum* ATCC13032 (Table S3). However, the differences in these genes were not statistically significant (*p* > 0.001); therefore, these 11 genes were not included in the list of 539 DEGs (Table S3). Previously reported results of genome sequencing revealed that in principle the genes encoding for all of the enzymes of the tricarboxylic acid cycle (TCA cycle), the glyoxylate cycle, and anaplerotic enzymes were present in *C*. *glutamicum* [11,12,13]. In our study, 10 out of 22 genes, which consist of 16 genes involved in the TCA cycle and 6 genes involved in anaplerotic reactions, were differentially expressed (*p* < 0.001) in *C*. *glutamicum* ATCC21300 compared to ATCC13032. Except for NCgl0659 (*pyc*, phosphoenolpyruvate carboxylase), which was downregulated, the other 9 genes (NCgl2167; *aceE*, NCgl0355; *lpd*, NCgl1482; *acn*, NCgl0634; *icd*, NCgl1084; *kgd*, NCgl2297; *mdh*, NCgl2765; *pck*, NCgl2008; *pyk*, and NCgl2521; *pqo*) were upregulated (Table S4).

**Figure 2:**
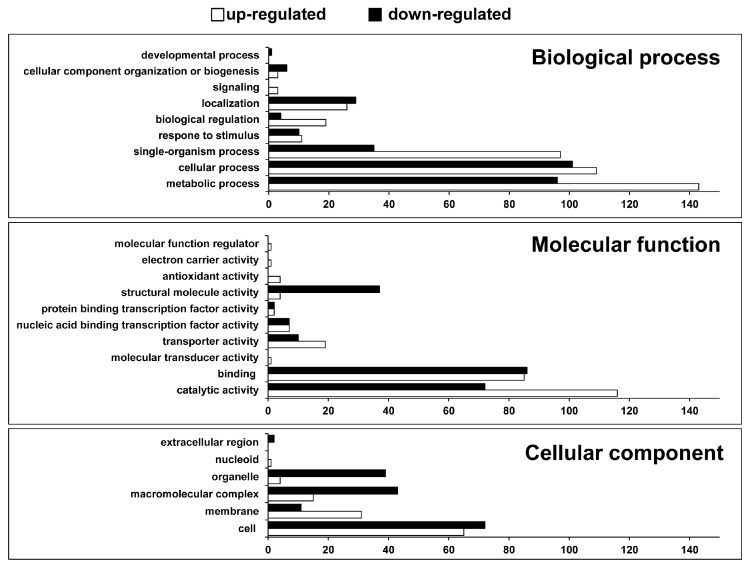
Functional categorization of up- and downregulated genes in *Corynebacterium glutamicum* ATCC21300 based on gene ontology (GO) annotations. Detailed information is shown in Table S5.

Using the Blast2GO software, which assigns biological functions based on BLAST sequence homologies and GO annotations (with respect to biological processes, molecular functions, and cellular components), 454 out of the 543 DEGs were assigned a biological function and categorized accordingly [14]. The biological processes mediated by these genes included “metabolic processes,” “cellular processes,” “single-organism processes,” *etc.* Their molecular functions were primarily related to “catalytic activity,” “binding activity,” *etc.* The cellular components involved encompassed the cell membrane, macromolecular complex, organelle, *etc.* Twice as many genes that were downregulated than were upregulated were associated with most categories, including “structural molecular activity,” “organelle,” and “macromolecular complex”; whereas a larger proportion of genes that were upregulated than were downregulated was associated with “metabolic process,” “biological regulation,” “transporter activity,” “catalytic activity,” and “single-organism process” (Figure 2 and Table S5).

We also determined GO terms that were enriched in the set of 543 DEGs (*p* < 0.001) using Blast2GO. A total of 6 and 8 GO terms, including “structural constituent of ribosome” (GO:0003735), “biotin biosynthetic process” (GO:0009102), were significantly enriched in the set of down- and upregulated genes, respectively (Table S6). Interestingly, among the significantly upregulated DEGs, NCgl0071 (encoding biotin synthase) expression was 20-fold higher (4.23 log2 fold change in reads per kilobase per million mapped reads (RPKM)) in the ATCC21300 strain than in the ATCC13032 strain, and more than 4-fold higher than the average expression (RPKM) level of all genes in the ATCC21300 strain (Table S7). In addition, NCgl2516 (*bioD*, encoding dithiobiotin synthetase) expression was significantly higher (2.29 log2 fold change in RPKM) in the ATCC21300 strain than in the ATCC13032 strain (Table S7).

### 2.2. Expression Patterns of Genes Involved in Biotin Metabolism

**Figure 3:**
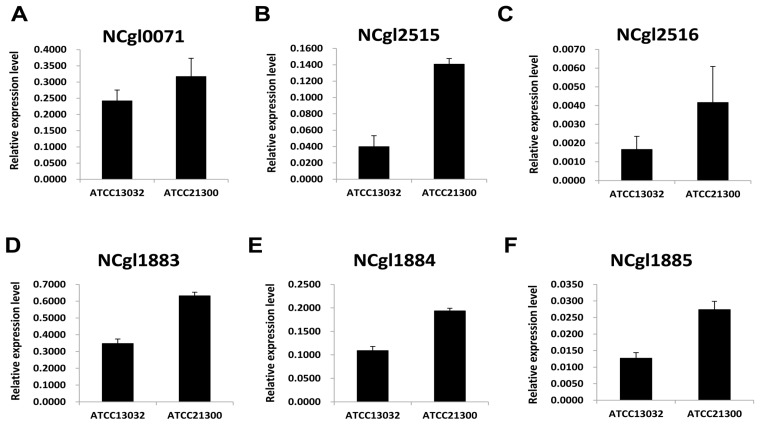
The expression patterns of genes associated with biotin synthesis and transport in *Corynebacterium glutamicum* ATCC13032 and ATCC21300. (**A**) The expression pattern of *bioB*; (**B**) the expression pattern of *bioA*; (**C**) the expression pattern of *bioD*; (**D**) the expression pattern of the *bioY* homolog; (**E**) the expression pattern of the *bioM* homolog; and (**F**) the expression pattern of the ABC transporter permease. The gene expression level (arbitrary units) was normalized using the 16s RNA level as an internal reference. Gene expression levels were quantified by real-time RT-PCR.

The number of DEGs involved in biotin synthesis and transport were different between the two strains based on GO enrichment analysis (Table 1 and Figure 3). Three genes (*bioA*, *bioD*, and *bioB*) were previously identified in *C*. *glutamicum* by complementation of *Escherichia coli* [15]. In this study, we found that the expression of these genes (*bioA*, *bioD*, and *bioB*) were 2.29-fold higher in ATCC21300 than in ATCC13032 (Table 1 and Figure 3). Interestingly, three genes (NCgl1883, NCgl1884, and NCgl1885) involved in biotin transport were also significantly upregulated in ATCC21300 compared to ATCC13032 (Table 1 and Figure 3).

### 2.3. Effects of bioB Mutation on l-Lysine Production, Growth Rate, and Biotin Transport-Related Gene (NCgl1883, NCgl1884, and NCgl1885) Expression

**Figure 4:**
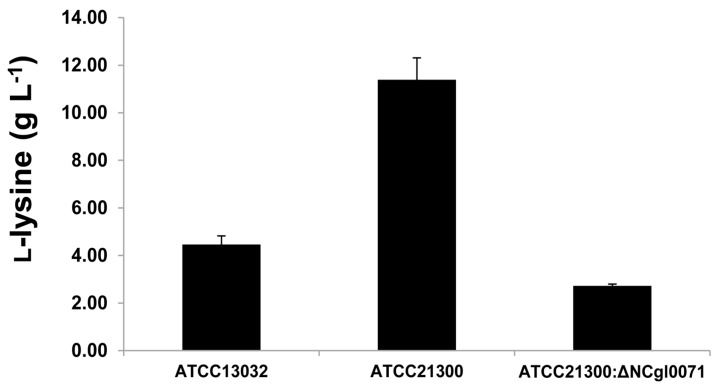
l-Lysine production of *Corynebacterium glutamicum* ATCC13032, ATCC21300, and the *bioB* mutant (of ATCC21300). Values shown are averages based on results obtained from at least three independent experiments, in which the standard deviation was <5%.

To evaluate whether l-lysine production and *bioB* gene upregulation were correlated, l-lysine production in ATCC21300 and in its *bioB* deficient mutant strain was analyzed by high performance liquid chromatography (HPLC). As shown in Figure 4, mutation of the *bioB* gene resulted in a ~76% decrease in l-lysine production. The average concentrations of l-lysine in the culture media of ATCC13032, ATCC21300, and the *bioB* deficient mutant were 4.47, 11.39, and 2.72 g·L^−1^, respectively (Figure 4).

**Figure 5:**
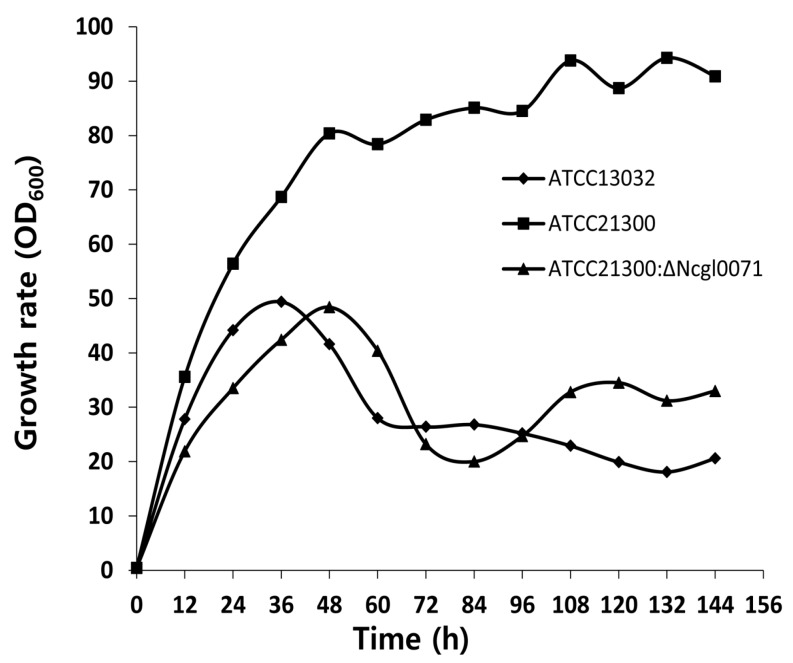
Growth rate of *Corynebacterium glutamicum* ATCC13032, ATCC21300, and the *bioB* mutant of ATCC21300.

To assay for any potential *in vivo* function of *bioB* (NCgl0071), growth rates of ATCC13032, ATCC21300, and the *bioB* mutant of ATCC21300 were analyzed. As shown in Figure 5, the growth rate of the *bioB* deficient mutant strain was severely decreased. The optical density of ATCC21300 strain became comparable with that of ATCC13032 and the *bioB* mutant of ATCC21300 after 36 h of growth. However, the growth rate of the *bioB* deficient mutant strain was similar to that of ATCC13032 strain, indicating that the mutation in the *bioB* gene severely affected the bacterial growth of ATCC21300 strain. This result also suggests that the *bioB* gene is not an essential gene for growth, although it is required for optimal growth rate ATCC21300 strain.

**Figure 6:**
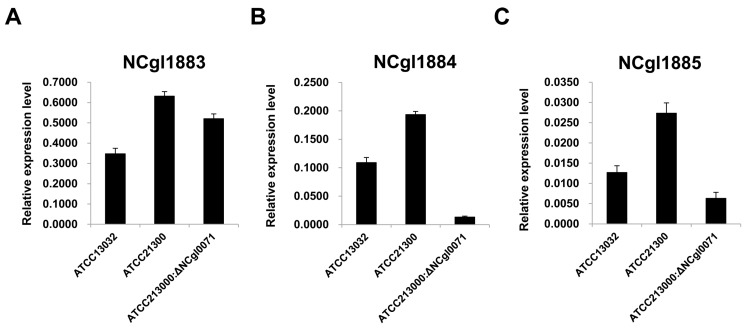
The expression patterns of genes associated with biotin transport in *Corynebacterium glutamicum* ATCC13032, ATCC21300, and the *bioB* mutant (of ATCC21300). (**A**) The expression pattern of the *bioY* homolog; (**B**) the expression pattern of the *bioM* homolog; (**C**) the expression pattern of the ABC transporter permease. The gene expression level (arbitrary units) was normalized using the 16s RNA level as an internal reference. Gene expression levels were quantified by real-time RT-PCR.

We investigated whether *bioB* (NCgl0071) gene expression was correlated with the gene expression of biotin transport-related genes, including NCgl1883, NCgl1884, and NCgl1885, using qRT-PCR analysis. As shown in Figure 6, two genes (NCgl1884 and NCgl1885) were expressed at relatively lower levels in the *bioB* deficient mutant than in either ATCC13032 or ATCC21300. Although the expression level of the NCgl1883 was relatively higher in the *bioB* deficient mutant than in ATCC13032, these results indicate that mutation of the *bioB* gene (NCgl0071) affects the expression of biotin transport-related genes, including NCgl1884 (encoding the ATP-binding proteins of an ABC carrier) and NCgl1885 (encoding the permease of an ABC transporter).

## 3. Discussion

Since the first use of *C. glutamicum* to commercially produce l-lysine, various approaches to improve the production of l-lysine were investigated. These approaches can be divided into the following three phases.

First, as described earlier, soon after it was discovered that *C. glutamicum* could be used to commercially produce amino acids, specific amino acid auxotroph mutants were discovered and used. The *C*. *glutamicum* ATCC13287 strain, which is auxotrophic for homoserine, produced l-lysine with conversion yields up to 26%. Kyowa Hakko reported a batch process using the *C*. *glutamicum* strain ATCC21300 (auxotrophic for threonine and leucine) that resulted in the production of 53.2 g·L^−1^ of l-lysine-HCl, which represented a 29% conversion yield [1]. During this phase, the major limitation of l-lysine producing strains was due to feedback inhibition by a mixture of l-lysine analogues *S*-(2-aminoethyl) cysteine and l-threonine [16]. Among the various enzymes, a feedback-resistant aspartate kinase (encoded by *lysC*) was shown to be one of the most important characteristics of l-lysine producing strains [1]. Thus, the development of strains auxotrophic for additional amino acids and vitamins was pursued, which led to the accumulation of a remarkable number of mutations. However, the additional nutrient requirements of auxotrophic strains obtained by random mutagenesis resulted in several drawbacks, including a greater sensitivity to higher temperatures or unfavorable pH. The drawbacks of auxotrophic strains eventually led to the development of leaky strains. This included a homoserine-leaky l-lysine producing strain, which was leaky because of reduced homoserine dehydrogenase activity [1]. Kelle *et al.* [1] also found that the homoserine dehydrogenase gene (NCgl1136) was slightly downregulated (−0.06 log2 fold change) in *C*. *glutamicum* ATCC21300 than in *C*. *glutamicum* ATCC13032 (Table S2).

The second phase was characterized by the rational development of strains using genetic engineering to specifically improve the biosynthetic pathways of randomly mutated strains. These strategies usually involved introducing feedback-resistant biosynthetic genes or upregulating the expression of extant feedback-resistant biosynthetic genes such as *lysC*. As described above, the feedback-resistant aspartate kinase (encoded by the *lysC* gene) was considered as a key enzyme for l-lysine production in *C*. *glutamicum* during the initial stages of its development as a l-lysine producing strain [1]. In addition to upregulating the feedback-resistant aspartate kinase, overexpression of the *lysE* gene (encoding lysine efflux permease) and *pyc* gene (encoding pyruvate carboxylase) enhanced l-lysine production by 500% and 50%, respectively [10,17]. However, our results indicated that expression of the *lysE* (NCgl1214) and *pyc* gene (NCgl0659) were lower in ATCC21300 than in ATCC13032 (Tables S2 and S3).

Current approaches to improve strains, which characterize the third phase, are more sophisticated and consider more than individual reactions in specific pathways. The goals of modern approaches involve optimizing the pH and temperature, as well as minimizing CO_2_ formation to increase the efficiency of respiration and reducing futile cycling. Ohnishi *et al.* [18] demonstrated that mutant strains that derived from genome breeding maintained their ability to produce l-lysine up to 40°C. This study also demonstrated complex changes in central metabolic pathway gene expression to different temperatures. This result suggests that improving l-lysine production, even in adverse conditions, will require a more detailed understanding of the regulatory networks in *C*. *glutamicum*. As described earlier, 10 out of 22 genes involved in the TCA cycle and 6 genes involved in anaplerotic reactions showed significantly different expression levels in *C*. *glutamicum* ATCC21300 compared with those of *C*. *glutamicum* ATCC13032 (Table S4). Redirection of the central metabolic pathway are considered crucial targets for strain development [10,19,20]. Peters-Wendisch *et al.* [10] reported that pyruvate carboxylase (*pyc* gene, NCgl0659) involved in the anaplerotic enzymes is a major bottleneck for glutamate and lysine production by *C*. *glutamicum* [10]. Thus, the key drivers of this phase are the availability of genome-wide analysis of wild-type and conventional production strains in addition to well-established post-genomics technologies.

Biotin is of particular interest since biotin auxotrophy led to the discovery that *C*. *glutamicum* can produce glutamate [21,22,23]. In addition, a sufficient amount of biotin is required for the production of certain amino acids, such as lysine and arginine, and is essential for *C*. *glutamicum* growth. However, the biotin biosynthesis pathway in *C*. *glutamicum* is incomplete due to the absence of the *bioF*, *bioW*, and *bioI* genes, which are involved in the de novo synthesis of pimeloyl-CoA. Although lacking the *bioF*, *bioW*, and *bioI* genes, the enzymes involved in biotin ring assembly (encoded by *bioA*, *bioD*, and *bioB*) are present in *C*. *glutamicum* [24]. It remains unclear how biotin enters the cell, since *C*. *glutamicum* is auxotrophic for biotin. Entcheva *et al.* [25] reported that the *bioM* gene appears to be related to biotin transport in *Sinorhizobium meliloti*. In *C*. *glutamicum*, a similar gene (NCgl1884) is present, which encodes the ATP-binding proteins of an ABC carrier. In addition, the gene (NCgl1885) encoding the permease of an ABC transporter and *bioY* (NCgl1883), which is involved in the bioconversion of pimelate into dethiobiotin, are positioned upstream and downstream of the *bioM* gene, respectively. In this study, transcriptome and qRT-PCR analyses revealed that the expression of 6 genes involved in either biotin biosynthesis (Ncgl0071, Ncgl2515, and Ncgl2516) or biotin transport (Ncgl1883, Ncgl1884, and Ncgl1885) was higher in ATCC21300 strain than in that of ATCC13032 (Figure 3, Table 1). Interestingly, the expression of these 6 genes was also downregulated when the *bioB* (NCgl0071) gene was disrupted (Figure 6). Furthermore, mutation of the *bioB* gene resulted in a ~76% decrease in l-lysine production (Figure 4). As shown in Figure 5, the *C*. *glutamicum* ATCC21300 strain had a different growth patterm when compared to that of ATCC13032 and the *bioB* deficient mutant strain. Thus, it was concluded that decreased lysine production by the *bioB* deficient mutant strain might be due to the effect of mutation in the *bioB* gene on impairment of the growth and on transcriptional regulation of other biotin related genes including Ncgl1883, Ncgl1884, and Ncgl1885. Indeed, Ko and Chipley [26] reported that increasing the biotin supply improved l-lysine production by *C*. *glutamicum* ATCC21806. They also suggested that biotin increased 14C-glucose uptake and affected fatty acid composition of cell wall lipids. Thus, they concluded that increased lysine production might be due to the stimulatory effect of biotin on the growth of *C*. *glutamicum*. Brune *et al.* [27] reported that the *bioQ* (Ncgl2025) gene (encoding a transcription regulator of the TetR protein family) acts as a repressor of genes involved in biotin metabolism or transport, such as *bioA*, *bioB*, *bioD*, *bioY* and *bioM*. However, there was no significant difference in expression level between the two strains (Table S1). We also previously identified genome-wide mutations in the *C*. *glutamicum* ATCC21300 genome by comparative analysis with the *C*. *glutamicum* ATCC13032 genome [28]. However, mutation including SNP and InDel was not detected in the *bioB* (NCgl0071), *bioY* (NCgl1883), Ncgl1884, Ncgl1885, *bioA* (NCgl2515), *bioD* (NCgl2516), and *bioQ* (NCgl2025) [28].

In the next decade, multidimensional approaches, including metabolic engineering and functional genomics, will be required to continue to develop industrial strains that will eventually replace current production strains developed by random mutagenesis and selection. Although it is unclear how disruption of the *bioB* gene affects biotin transport-related gene expression, decreased expression of biotin transport-related genes may account for the reduced production of l-lysine in the *bioB* deficient mutant strain. Our findings are the first to suggest that expression of the NCgl0071 (*bioB*, encoding biotin synthase) gene in *C*. *glutamicum* affects l-lysine production and the co-expression of biotin transport-related genes. Although further studies are required to elucidate the relationship between the *bioB* gene and biotin transport-related genes, our findings should facilitate the identification of beneficial genes from each genome strain and will provide valuable insights for future strategies of metabolic engineering to industrially produce amino acids at high levels.

## 4. Materials and Methods

### 4.1. Bacterial Culture and Total RNA Isolation

The *C*. *glutamicum* strains, ATCC21300 and ATCC13032, were obtained from the Korean Collection for Type Culture (KCTC) in Daejeon, Korea, and maintained in brain-heart infusion (BHI) medium (DB, Seoul, Korea) at 30 °C. *Escherichia coli* DH5a (Thermo Scientific, Seoul, Korea) cells were grown in Luria–Bertani (LB) (DB, Seoul, Korea) broth or on LB-agar plates for all recombinant DNA experiments that required a bacterial host. For l-lysine production experiments and total RNA isolation, a seed culture was prepared by inoculating cells into recovery medium (80 g BHI, 60 g sorbitol and 20 g glucose, and per liter) and growing the cells overnight. Cells were harvested, resuspended, and incubated in a 100-mL baffled Erlenmeyer flask containing 10 mL of MMY medium (0.8 g KH_2_PO_4_, 10 g (NH_4_)_2_SO_4_, 1 g MgSO_4_·7H_2_O, 1.2 g Na_2_HPO_4_, 2 mg MnSO_4_·H_2_O, 2 mg FeSO_4_·7H_2_O, 1 mg ZnSO_4_·7H_2_O, 10 g yeast extract, and 60 g glucose per liter (pH 7.0)), followed by cultivation at 30 °C until the stationary phase. Total RNA was extracted from *C. glutamicum* following methods described by Jahn *et al.* [29]. Total RNA was further treated with the RNase-Free DNase Set (Qiagen, Seoul, Korea) according to the manufacturer’s instructions. Total RNA was analyzed for its integrity and quality (value greater than or equal to 8) using an Agilent Technologies 2100 Bioanalyzer (Agilent Technologies, Santa Clara, CA, USA).

### 4.2. Illumina Sequencing and Raw Data Analysis

Next-generation sequencing (NGS) sequencing was performed with 1 μg of total RNA using the HiSeq2000 platform according to the manufacturer’s protocol (Illumina, Inc., San Diego, CA, USA). Illumina Casava (version 1.8.0) (Illumina, Inc.) was used for base calling, and all sequencing data were processed for further analysis by trimming bad quality reads (FASTX-Toolkit version 0.0.13; http://hannonlab.cshl.edu/fastx_toolkit/) and removing sequencing adapters [30]. All the tags were used in the following steps. Short reads were directly mapped to reference sequences using BWA (Burrows–Wheeler Aligner) [31] with the parameter set at −q 20 (threshold quality for read trimming). Mapped reads were further processed by SAMtools [32], and the expression level was evaluated by the reads per kilobase per million mapped reads (RPKM). DEGseq (two class unpaired MA-plot-based method) [33] was used to detect differentially expressed genes between two samples. *p* < 0.001 was considered significant. The genome sequence of *C*. *glutamicum* was obtained from the National Center for Biotechnology Information (NCBI, www.ncbi.nlm.nih.gov) database. RNA-seq data were deposited in the NCBI database under accession numbers SRP021063 (*C*. *glutamicum* ATCC21300) and SRP021064 (*C*. *glutamicum* ATCC13032). GO term annotation (molecular function, biological process, and cellular component) and enrichment analysis of a subset of DEGs (*p* < 0.001) was conducted using the Blast2GO software (version 3.0) with default parameters [14].

### 4.3. Validation of Differentially Expressed Genes (DEGs) Using Quantitative Real Time PCR

Total RNA samples were prepared following the same protocol as for the Illumina analysis. The sequence of each gene in the *C. glutamicum* ATCC13032 database (NC_003450, http://www.ncbi.nlm.nih.gov) was used for designing primers (Integrated DNA Technologies INC, Coralville, IA, USA). The reaction mixture contained: 25 ng of cDNA, 5 pmol of each primer (Table S1), a 12.5-µL SensiFAST SYBR No-ROX kit buffer (Bioline, Alexandria, Australia) and DEPC-treated water up to 25 μL. Quantitative real-time PCR was performed in a RotorGene 6000 (Qiagen) using the thermocycler program: 95 °C for 3 min followed by 40 cycles for 5 s at 95 °C, 10 s at 60 °C, and 15 s at 72 °C.

### 4.4. Site-Specific Gene Disruption

To construct a *C. glutamicum* mutant strain encoding an internally deleted NCgl0071 (*bioB*, encoding biotin synthase), DNA sequences in the genome of *C*. *glutamicum* ATCC21300 located upstream and downstream of the ORF, NCgl0071, were amplified by PCR using the following primer pairs: bioBUF (5′-CAAGGCCGTTCAACCGCGCT-3′) and bioBUR (5′-AAGCTTTCTGTTTAGCGGCTTCAACC-3′; *HindIII* restriction site is underlined), and bioBDF (5′-AAGCTTATAATTTGGAAACTGCGCGT-3′; *HindIII* restriction site is underlined) and bioBDR (5′-TTAGATGACCTTATTAAGGA-3′), respectively. PCR products were digested with *HindIII* and ligated into the pGEM-T-Easy vector (Promega, Seoul, Korea). The resulting plasmid was digested with *EcoRI*, and the internally deleted *bioB* gene fragment was ligated into the corresponding sites of pK18mobsacB [34]. *C*. *glutamicum* ATCC21300 was transduced with the resulting plasmid via electroporation, and the mutant strain was created using a method described previously by Yoon *et al.* [35].

### 4.5. High Performance Liquid Chromatography Analysis of l-Lysine Production

Cell growth in MMY broth (0.8 g KH_2_PO_4_, 10 g (NH_4_)_2_SO_4_, 1 g MgSO_4_∙7H_2_O, 1.2 g Na_2_HPO_4_, 2 mg MnSO_4_∙H_2_O, 2 mg FeSO_4_∙7H_2_O, 1 mg ZnSO_4_∙7H_2_O, 10 g yeast extract, and 60 g glucose per liter (pH 7.0)) was estimated by measuring OD_600_ using a spectrophotometer. l-lysine concentrations (g·L^−1^ culture medium) were determined using HPLC (Shimadzu, Kyoto, Japan) and a SUPELCOSIL™ LC-18-DB HPLC column (4.6 mm × 250 mm, 5 μm; Sigma-Aldrich, Yongin-si, Korea). A gradient of 100:0 A:B to 0:100 A:B for 0–50 min at a flow rate 0.9 mL·min^−1^ was used. The concentration of l-lysine was quantified using a standard curve (Bio basic INC, Seoul, Korea).

### 4.6. Growth Rate Analysis

For growth rate assay, single colonies of each strain were separately inoculated in a recovery medium (80 g BHI, 60 g sorbitol and 20 g glucose, and per liter) and incubated for 144 h at 30 °C in a shaking incubator. Fifty microliters of each culture (OD_600_ = 0.4) were inoculated in 10 mL of an MMY medium (0.8 g KH_2_PO_4_, 10 g (NH_4_)_2_SO_4_, 1 g MgSO_4_·7H_2_O, 1.2 g Na_2_HPO_4_, 2 mg MnSO_4_·H_2_O, 2 mg FeSO_4_·7H_2_O, 1 mg ZnSO_4_·7H_2_O, 10 g yeast extract, and 60 g glucose per liter (pH 7.0)). The optical desities (ODs) of each strain were measured every 12 h using the Genesys-20 spectrophotometer (Thermo Scientific, Seoul, Korea).

## Figures and Tables

**Table 1:** Expression patterns of genes involved in biotin metabolism in *Corynebacterium glutamicum* ATCC13032 and ATCC21300.

Locus ID	Gene Name	Product	RPKM *		log2 Fold Change	Signature (*p*-value < 0.001)
			ATCC21300	ATCC13032		
NCgl2515	*bioA*	adenosylmethionine-8-amino-7-oxononanoate aminotransferase	200.53	11.49	4.08	TRUE
NCgl2516	*bioD*	dithiobiotin synthetase	264.71	52.29	2.30	TRUE
NCgl0071	*bioB*	biotin synthase	1522.14	77.71	4.25	TRUE
NCgl1883	*bioY* homolog	hypothetical protein	858.40	35.04	4.57	TRUE
NCgl1884	*bioM* homolog	ABC transporter ATPase	751.37	60.68	3.59	TRUE
NCgl1885	-	ABC transporter permease	322.68	61.30	2.36	TRUE

* RPKM: reads per kilobase per million mapped reads

## Supplementary Materials

Supplementary materials can be found at http://www.mdpi.com/1422-0067/17/3/353/s1.

## Abbreviations

DEGs: differentially expressed genes
GO: gene ontology
NGS: next-generation sequencing
qRT-PCR: quantitative real-time RT-PCR
RPKM: read per kilobase per million mapped reads
TCA cycle: tricarboxylic acid cycle
